# ERA Registry Figure of the month Time trends in dialysis incidence across age groups and countries

**DOI:** 10.1093/ckj/sfaf013

**Published:** 2025-01-20

**Authors:** Vianda S Stel, Alberto Ortiz, Anneke Kramer

**Affiliations:** ERA Registry, Department of Medical Informatics, Amsterdam UMC – Location University of Amsterdam, Amsterdam, the Netherlands; Amsterdam Public Health Research Institute, Quality of Care, Amsterdam, the Netherlands; Department of Nephrology and Hypertension, IIS-Fundacion Jimenez Diaz UAM, Madrid, Spain; Department of Medicine, Universidad Autonoma de Madrid, Madrid, Spain; ERA Registry, Department of Medical Informatics, Amsterdam UMC – Location University of Amsterdam, Amsterdam, the Netherlands; Amsterdam Public Health Research Institute, Quality of Care, Amsterdam, the Netherlands

**Figure 1:**
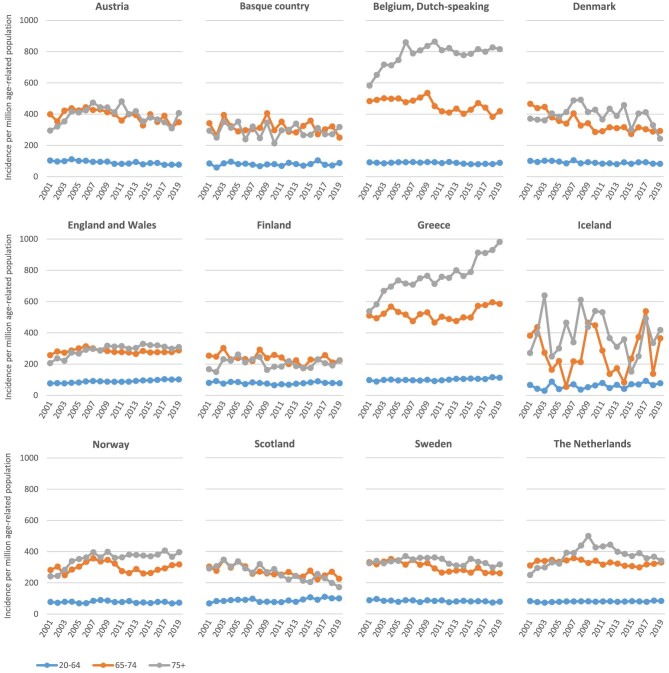
Trends in dialysis incidence at day 91 between 2001 and 2019, by age group and country.**Source**: Van Oevelen et al. al. CKJ 2023, https:/doi.org/10.1093/ckj/sfad048, Fig. 2.**Explanation**: The number of patients starting dialysis per million age-related population (pmarp) for those aged 75 years and older was already highest in Belgium and Greece at the beginning of this century and has increased mainly in these countries thereafter. In several other counties, like Denmark, Scotland and the Netherlands, there was a decrease in the number of older patients starting dialysis pmarp during the last decade.

